# Exposure to Crime at Food Stores: Implications for Nutrition and Health among Black Americans

**DOI:** 10.1007/s10900-024-01436-4

**Published:** 2025-01-20

**Authors:** Chelsea R. Singleton, Danielle J. Gartner, Fikriyah Winata, Donald Rose, Karen M. Sheehan, Sara L. McLafferty

**Affiliations:** 1 Department of Social, Behavioral, and Population Sciences, Celia Scott Weatherhead School of Public Health & Tropical Medicine, Tulane University, 1440 Canal Street, New Orleans, LA USA 70112; 2 Department of Geography, Texas A&M University, College Station, TX USA; 3 Departments of Pediatrics, Medical Education, and Preventive Medicine, Northwestern Feinberg School of Medicine, Chicago, IL USA; 4 Department of Geography and Geographic Information Science, University of Illinois at Urbana-Champaign, Urbana, IL USA

**Keywords:** Crime, Grocery store, Food insecurity, Obesity, Dietary behavior, Black communities

## Abstract

Crime is a public health issue that disproportionately affects racially-marginalized populations. Studies have reported that food stores (e.g., grocery stores, convenience stores) often attract crime due to their volume of cash transactions and limited security. Little is known about how exposure to crime at food stores affects nutrition or health. This study aimed to fill this research gap by exploring the lived experiences of Black Americans. In 2023, 502 Black-identifying adults completed a survey online. They reported their socio-demographics, fruit and vegetable (FV) consumption, food security status, height, weight, and experiences with crime at food stores in their community. Multivariable-adjusted regression models were examined to identify associations between exposure to crime at food stores and the following measures: low food security status, obesity status, and daily servings of FVs. Approximately 150 (29%) participants avoided one or more food stores in their community due to crime; 102 (20%) had witnessed a crime at a food store. Those who avoided food stores had greater odds of low food security (OR: 1.94; 95% CI: 1.25–3.02) and obesity (OR: 2.15; 95% CI: 1.33–3.48) compared to others. Those who witnessed a crime had greater odds of low food security (OR: 3.14; 95% CI: 1.82–5.41). Exposure to crime at food stores was not associated with FV consumption after adjusting for socio-demographics. Exposure to crime in food stores may have negative health and nutritional implications. Future studies should explore these implications for populations that are disproportionately affected by crime.

## Introduction

For decades, scholars have documented the negative health effects of crime exposure [1–3]. Residing in a community with high crime rates, especially violent crime rates (e.g., homicide, armed robbery), increases risk of adverse health outcomes such as substance abuse, stress, and depression [4, 5]. Crime exposure can also increase risk of chronic diseases (e.g., obesity, type 2 diabetes, cardiovascular disease) because exposure often reduces engagement in relevant health behaviors such as leisure-time physical activity, walking, and public park use [6–10].

The evidence connecting crime exposure to chronic disease risk is concerning given the racialized disparities in exposure that exist in the U.S [11–14]. Historically racialized communities often have higher crime rates compared to White communities, and racially-marginalized adults and youth are more likely to witness a crime or be victimized in their lifetime compared to their White counterparts [13, 14]. These disparities emphasize the need to improve understanding of exposure to crime among racially-marginalized populations and the implications for health and well-being.

Although U.S. adults are primarily exposed to crime and violence in their own home, the Centers for Disease Control and Prevention reports that low-income, Black, and Latino populations are disproportionately exposed to crime occurring between two non-related individuals while in their community’s public spaces [15, 16]. These spaces include schools, parks, recreation centers, and even food stores (e.g., grocery stores) [15, 16]. Studies have shown that food stores are highly susceptible to crime because of their hours of operation, cash transaction volume, and limited security [17, 18]. Crime rates are higher in communities with greater availability of certain food stores, such as convenience stores, dollar stores, liquor stores, and fast-food restaurants [19–23]. Furthermore, food stores, particularly convenience stores and liquor stores, often appear on the Federal Bureau of Investigation’s annual list of business formats where the most violent crimes occur in the U.S [19]. This evidence implies that food stores play an important role in the risk of crime exposure.

To date, studies examining exposure to crime at food stores have solely focused on outcomes such as injury and death [24, 25] Exposure to crime at food stores may have nutritional consequences that are relevant to chronic disease risk. For example, exposure to crime at food stores may negatively influence an individual’s food shopping behavior and perceptions of food access in their community, which, according to previous studies, are associated with food security and diet quality [26–28]. To generate evidence that supports this theory and expand current knowledge of crime exposure at retail businesses, we explored self-reported exposure to crime at food stores among Black-identifying U.S. adults to determine if exposure was associated with food security, obesity, or daily fruit and vegetable (FV) consumption. We hypothesized that participants who self-report exposure to crime at food stores in their community will have lower FV consumption and higher odds of food insecurity and obesity.

## Methods

### Study Design and Participants

We conducted a cross-sectional survey in 2023 via the online survey platform– *Cloud Research* [29]. Cloud Research connects researchers to prospective survey respondents by drawing specialized research panels from the 2 + million U.S. residents registered on their platform [29]. Our research team designed an online survey using Qualtrics software to collect important information on exposure to crime at food stores and several nutritional and health indicators.

After finalizing the survey, Cloud Research staff distributed it to all registered survey takers who met our eligibility criteria: age 18 or older, self-identify as Black or African American, lives in the U.S. The survey link was active for 3 weeks in October 2023; during this time, eligible adults were able to complete the survey. Those who opted to complete the survey were compensated. After the survey period, we extracted the data from Qualtrics and performed a thorough quality control review with Cloud Research staff. Of the 601 survey records captured, only 502 (84%) were included in the final study sample. We excluded 24 records because the respondent listed their race/ethnicity as something other than Black/African American. We excluded 75 records because the respondent did not answer < 50% of the survey. This study was approved by the Institutional Review Board at Tulane University.

### Variable Definitions

#### Exposure to Crime at Food Stores

Our two independent variables were *(1)* avoidance of food stores due to crime and *(2)* witnessing a crime at a food store. Respondents were asked to indicate (yes or no) if they avoided one or more food stores in their community due to crime. Examples of crime provided on the survey include homicide, armed robbery, shootings, drug sales, gang activity, and theft. If they responded “yes”, they were prompted to indicate the store format for the food store(s) they avoided: grocery store/supermarket, supercenter (example: Wal-Mart), Club Store (example: Costco), corner store (including convenience stores, gas stations, and bodegas), dollar store (example: Dollar General), drug store (example: Wal-Greens), liquor store, fast-food restaurant, full-service/dine-in restaurant, or farmers market/farm stand. Respondents were allowed to check all store types that applied. Afterwards, respondents were asked to indicate (yes or no) if they had ever witnessed a crime at a food store in their community. They were given the same examples of crime for this question and prompted to list the relevant store format(s) if they answered yes to the question.

#### Food Security, Obesity, and Fruit and Vegetable Consumption

We examined three dependent variables: food security status, obesity status, and daily FV consumption. To measure food security status, participants completed the 6-item U.S. Food Security Module developed by the U.S. Department of Agriculture [30]. This module is a valid and reliable tool for measuring household food security status in the prior 30 days [30]. Respondents’ answers were scored to determine if they were food secure (score = 0) or experiencing marginal security (score = 1), low food security (score = 2–4) or very low food security (score = 5–6). All respondents were asked to self-report their current height (in feet and inches) and weight (in pounds). We used these estimates to calculate their body mass index (BMI) according to the formula weight (lb)/[height (in)]^2^ × 703 [31]. Respondents with a BMI ≥ 30 were labeled obese.

Respondents were prompted to complete a modified version of the Dietary Screener Questionnaire (DSQ) to measure their daily FV consumption [32]. The DSQ is a valid and reliable tool developed for the National Health & Nutrition Examination Survey (NHANES) that can screen for daily intake of several foods [32, 33]. For this study, respondents completed the screener items used to calculate daily FV consumption: % fruit juice, fruit, salad, other vegetables, fried potatoes, other potatoes, beans, salsa, and tomato sauce. For each item, respondents indicated their frequency of consumption in the prior 30 days: never, 1 time last month, 2–3 times last month, 1 time per week, 2–3 times per week, 3–4 times per week, 5–6 times per week, 1 time per day, 2 or more times per day. We used the corresponding SAS program and scoring algorithm to determine each respondent’s daily intake of FVs in cups [33].

#### Socio-Demographic Characteristics

We evaluated several socio-demographic characteristics as covariates. This includes age (years), sex (female, male, or non-binary), ethnicity (Hispanic or non-Hispanic), marital status (married or not married), education level (≤ high school, some college, or ≥ bachelor’s degree), number of household members, Supplemental Nutrition Assistance Program (SNAP) participation status (yes or no), and residential location (large city, small city, town, or rural area). For education level, the “some college” category included respondents that had an associate degree or vocational degree. The “bachelor’s degree” category included respondents with a graduate or professional degree. For SNAP participation status, respondents answered yes or no if they, or a member of their household, received SNAP benefits in the last 12 months. For residential location, a note was added in the survey to let respondents know that a large city has > 100,000 residents.

### Statistical Analysis

We calculated descriptive statistics (means and frequencies) for our variables among all survey respondents and stratified by our two independent variables: avoidance status (yes or no) and witness status (yes or no). We ran chi-square tests of independence (categorical variables) and *t* tests (continuous variables) to determine if the variables were associated with avoidance status or witness status. We ran multivariable-adjusted logistic regression models to determine if avoidance status or witness status were associated with odds of low food security or obesity when controlling for age, sex, ethnicity, marital status, education level, household size, SNAP participation status, and residential location. Only 432 respondents provided valid estimates of their body composition, so the sample for the obesity models was limited to 432. In the logistic regression models, we defined “low food security status” as experiencing low or very low food security. We ran multivariable-adjusted linear regression models to determine if avoidance status or witness status were associated with servings of FVs consumed per day (cups). These models were adjusted for the same covariates included in the logistic regression models. We considered *P* values greater than 0.05 to be significant and used SAS 9.4 to perform the analyses [34].

## Results

Descriptive characteristics of respondents are presented in Table 1 stratified by avoidance status and witness status. Approximately 150 participants (29.9%) avoided one or more food stores in their community due to crime; 102 (20.5%) witnessed a crime at a food store in their community. Mean age was 47.2 years old and 60.2% of respondents were female. Black Hispanic adults represented 6.2% of respondents, 33.4% of respondents were married, and 32.0% had at least a bachelor’s degree. Most reported living in a large city (75.3%) and mean number of household members was 2.6. Approximately 49.2% of respondents were experiencing low or very low food security, and 39.5% were SNAP participants. Mean FV intake per day was 2.3 cups, and 33.3% were obese according to their BMI.

**Table 1:** Descriptive characteristics of survey respondents stratified by avoidance status & witness status

Variable:		Avoids Food Stores Due to Crime			Witnessed a Crime at a Food Store		
	All Participants	Yes	No		Yes	No	
	*N* = 502	150 (29.9)	352 (70.1)	*P* Value^a^	102 (20.5)	396 (79.5)	*P* Value
*Demographics*							
Age (years), mean (sd)	47.2 (16.7)	41.2 (13.9)	49.8 (17.1)	< 0.0001	40.7 (14.2)	49.0 (16.9)	< 0.0001
Sex, n (%)				0.18			0.25
Female	302 (60.2)	97 (64.7)	205 (58.2)		56 (54.9)	242 (61.1)	
Other^b^	200 (39.8)	53 (35.3)	147 (41.8)		46 (45.1)	154 (38.9)	
Ethnicity, n (%)				0.06			0.0004
Hispanic	31 (6.2)	14 (9.3)	17 (4.8)		14 (13.7)	17 (4.3)	
Not Hispanic	471 (93.8)	136 (90.7)	335 (95.2)		88 (86.3)	379 (95.7)	
Marital Status, n (%)				0.69			0.46
Married	167 (33.4)	52 (34.7)	115 (32.9)		31 (30.4)	135 (34.3)	
Not Married	333 (66.6)	98 (65.3)	235 (67.1)		71 (69.6)	259 (65.7)	
Education Level, n (%)				0.11			0.21
≤High School/GED	140 (28.0)	45 (30.0)	95 (27.1)		23 (22.5)	116 (29.4)	
Some College^c^	200 (40.0)	67 (44.7)	133 (38.0)		48 (47.1)	150 (38.1)	
≥Bachelor’s Degree	160 (32.0)	38 (25.3)	122 (34.9)		31 (30.4)	128 (32.5)	
Residential Location, n (%)				0.18			0.47
Large City (≥ 100,000)	378 (75.3)	107 (71.3)	271 (77.0)		80 (78.4)	297 (75.0)	
Other^d^	124 (24.7)	43 (28.7)	81 (23.0)		22 (21.6)	99 (25.0)	
*Household*							
Number of Household Members, mean (sd)	2.6 (1.6)	2.9 (1.6)	2.5 (1.5)	0.003	3.1 (1.9)	2.5 (1.4)	0.005
SNAP Participation, n (%)				0.001			< 0.0001
Yes	198 (39.5)	75 (50.3)	123 (34.9)		60 (58.8)	136 (34.4)	
No	303 (60.5)	74 (49.7)	229 (65.1)		42 (41.2)	259 (65.6)	
Household Food Security, n (%)				< 0.0001			< 0.0001
Food Secure/Marginal Security	255 (50.8)	50 (33.3)	205 (58.2)		24 (23.5)	230 (58.1)	
Low Food Security	108 (21.5)	33 (22.0)	75 (21.3)		16 (15.7)	89 (22.5)	
Very Low Food Security	139 (27.7)	67 (44.7)	72 (20.5)		62 (60.8)	77 (19.4)	
*Nutrition & Health*							
Daily Servings of FV (cups), mean (sd)	2.3 (0.7)	2.2 (0.6)	2.4 (0.7)	0.005	2.2 (0.5)	2.4 (0.7)	0.04
Obesity Status, n (%)				0.009			0.68
Obese^e^	144 (33.3)	53 (42.7)	91 (29.6)		27 (31.4)	116 (33.7)	
Non-Obese	288 (66.7)	71 (57.3)	217 (70.4)		59 (68.6)	228 (66.3)	

FV: fruit and vegetables; SNAP: Supplemental Nutrition Assistance Program; sd: standard deviation

Note: Cells may not sum to total due to missing data.

a.) P Values calculated with t-test or chi-square test of independence.

b.) Other category for sex includes those who self-identified as male or non-binary.

c.) Some college category for education level includes those with an associate’s degree or vocational degree.

d.) Other category for place of residence includes participants who reported living in small cities, towns, and rural areas.

e.) Obesity is defined as a BMI ≥ 30.

Results from bivariate analyses indicated that avoidance status was significantly associated with age, number of household members, SNAP participation, food security, daily FV intake, and obesity. Compared to others, those who avoided one or more food stores in their community due to crime were, on average, younger, had more household members, and consumed fewer servings of FVs per day. In addition, they had a greater proportion of SNAP participants and a greater proportion of low food security and obesity. Witness status was significantly associated with age, ethnicity, number of household members, SNAP participation, food security, and daily FV intake. Compared to others, those who witnessed a crime at a food store in their community were, on average, younger and had a greater proportion of Hispanic participants and SNAP participation. They had a lower mean intake of FVs and a greater proportion of participants experiencing low food security.

Table 2 presents frequencies for each food store format mentioned by respondents who self-reported being exposed to crime at food stores. The four most common store formats listed the most by respondents who avoided one or more food stores in their community due to crime were grocery/supermarket (48.7%), convenience store (48.0), supercenter (32.7%), and dollar store (27.3%). Respondents who witnessed a crime at a food store selected convenience store (52.0%), grocery store/supermarket (37.3%), dollar store (27.5%), and supercenter (24.5%).

**Table 2:** Food stores reported by survey respondents that avoid food stores due to crime and witnessed a crime at a food store, n (%).^A^

Store Format:	Avoids Food Stores Due to Crime	Witnessed a Crime at a Food Store
	*N* = 150	*N* = 102
Grocery Store/Supermarket	73 (48.7)	38 (37.3)
Supercenter	49 (32.7)	25 (24.5)
Club Store	20 (13.3)	11 (10.8)
Convenience Store^a^	72 (48.0)	53 (52.0)
Dollar Store	41 (27.3)	28 (27.5)
Liquor Store	26 (17.3)	21 (20.6)
Drug Store/Pharmacy	20 (13.3)	21 (20.6)
Fast Food Restaurant	19 (12.7)	10 (9.8)
Full-Service Restaurant	7 (4.7)	1 (0.5)
Farmers Market/Farm Stand/CSA	12 (8.0)	2 (2.0)

CSA: community supported agriculture program

a.) Frequencies will not sum to 100% because survey respondents were allowed to select all formats that apply

b.) Convenience store includes bodegas, corner stores, and gas stations

Results from logistic regression models examining associations between exposure to crime at food stores, low food security status, and obesity status are presented in Table 3. After adjusting for covariates, those who avoided one or more food stores due to crime had greater odds of low food security compared to others (OR: 1.94; 95% CI: 1.25–3.02). Those who witnessed a crime at a food store also had greater odds of low food security status compared to others (OR: 3.14; 95% CI: 1.82–5.41). After adjusting for covariates, those who avoided food stores due to crime had greater odds of obesity compared to others (OR: 2.15; 95% CI: 1.33–3.48). Witness status was not associated with obesity.

**Table 3:** Logistic regression models examining associations between exposure to crime in food stores, low food security status, and obesity status

Variable	Low Food Security^a^		Obesity^b^	
	Crude OR (95% CI)	Adjusted OR (95% CI)^c^	Crude OR (95% CI)	Adjusted OR (95% CI)^c^
Avoids Food Stores Due to Crime^d^	2.79 (1.87–4.16)	1.94 (1.25–3.02)	1.78 (1.16–2.74)	2.15 (1.33–3.48)
Witnessed a Crime at a Food Store^e^	4.50 (2.73–7.42)	3.14 (1.82–5.41)	0.90 (0.54–1.49)	0.97 (0.56–1.68)

CI: confidence interval; OR: odds ratio

a.) Low food security includes those participants experiencing low and very low food security. Sample size for models = 502

b.) Obesity is defined as a BMI ≥ 30. Sample size for models = 432

c.) Model adjusted for age, sex, ethnicity, marital status, education level, household size, SNAP participation status, and place of residence

d.) Reference group for model is participants who do NOT avoid food stores in their community due to crime

e.) Reference group for model is participants who have NOT witnessed a crime at a food store in their community

Table 4 displays results from linear regression models examining associations between exposure to crime in food stores and daily FV intake. Crude models suggested that those who avoided stores due to crime and those who witnessed a crime at a food store consumed fewer cups of FVs per day compared to others. After adjusting for covariates, neither avoidance status nor witness status was associated with daily FV intake (*p* = 0.07 for both).

**Table 4:** Linear regression models examining associations between exposure to crime in food stores and daily FV intake

Variable:	FV Intake (Cups)^a^			
	Crude β (SE)	*P* Value	Adjusted β (SE)^b^	*P* Value
Avoids Food Stores Due to Crime^c^	-0.17 (0.07)	0.01	-0.12 (0.07)	0.07
Witnessed a Crime at a Food Store^d^	-0.16 (0.08)	0.04	-0.14 (0.08)	0.07

β: parameter estimate; FV: fruit and vegetables; SE: standard error

a.) Represent daily servings of fruit and vegetables in cup equivalents

b.) Model adjusted for age, sex, ethnicity, marital status, education level, household size, SNAP participation status, and place of residence

c.) Reference group for model is participants who do NOT avoid food stores in their community due to crime

d.) Reference group for model is participants who have NOT witnessed a crime at a food store in their community

## Discussion

We aimed to examine exposure to crime at food stores among Black-identifying adults to determine if exposure is associated with household food security, obesity, or FV consumption. The evidence documenting ways in which crime exposure at food stores hinders health and nutritional well-being is scarce [24, 25] Despite the scarcity of literature on this specific topic, several studies offer insight and context to our research findings [35–41].

We found that both variables representing exposure to crime at food stores increased odds of food insecurity among our survey respondents. These findings align with prior studies on the relationships between crime, violence, and food insecurity [34–37]. A mixed-methods study by Chilton and colleagues reported that early experiences of trauma and violence were correlated with food insecurity among mothers in Philadelphia, PA [35]. Ali and colleagues associated incidents of firearm injuries with food insecurity at the community level in New Orleans, LA [36]. Kaila and Azad linked crime victimization to food insecurity and decreased food consumption in conflict-affected areas of Nigeria [37]. A 2021 paper by Miller and colleagues describes the complex relationships between violence and food insecurity [38] They posited that violence and food insecurity, like healthy food access, are indicators of structural disadvantage, which explains their association [38]. To our knowledge, no study has established a theoretical pathway connecting exposure to crime at food stores with food shopping behaviors, perceptions of food access, and ultimately, food insecurity. This research is needed to provide context to our study findings.

We found some evidence that exposure to crime at food stores was associated with obesity. Only avoidance status increased odds of obesity. Several studies have linked crime to obesity [6, 8, 39]. Singleton and colleagues associated higher violent crime rates with higher prevalence of adult obesity among census tracts in Chicago, IL [8]. Chaparro and colleagues reported that exposure to neighborhood crime was associated with higher odds of overweight/obesity among adolescent girls participating in NHANES [39]. Exposure to crime, in general, is a predictor of physical activity engagement, which might explain our findings [8–10, 39]. However, there may be theorical pathways connecting exposure to crime at food stores to obesity that involves intermediate factors such as food security and geographic access to healthy foods [3]. Again, more research on this topic is needed to test these potential pathways.

Crude models suggested a negative association between exposure to crime at food stores and daily FV consumption. After adjusting for covariates, the associations were no longer significant. Although no prior study has examined exposure to crime at food stores in related to dietary behavior, some have examined exposure to domestic and community violence [40, 41]. Shuler and colleagues found that adverse childhood experiences, including domestic violence and parental incarceration, were associated with lower fruit intake in children [40]. Robles and colleagues reported an association between higher perceived community violence and lower FV consumption in a diverse sample of adults in Los Angeles, CA [41]. It is possible that our sample size was insufficient to detect a significant association between exposure to crime at food stores and FV consumption. Future research should consider this potential weakness as well as other aspects of diet other than FV consumption.

It is important to acknowledge that business and economics experts have a long-standing history of documenting the detrimental effects of crime on retail businesses, their employees, and their customers [42–45]. According to this literature, crime increases the cost of running a business since stores in crime-burdened areas often have to pay more for insurance, security measures, and product loss [42, 43]. Increased costs reduces the financial sustainability of a business and deters businesses from opening in a community [44, 45] Furthermore, crime can be physically and psychologically harmful to store employees and customers with studies reporting that employees and customers are at risk of serious injury and death when a crime is occurring at a retail business [24, 25, 43]. Overall, this research, and our study findings, are highly relevant to public health efforts to address structural inequities in food access among racialized communities [46, 47]. Crime should be considered in future research and initiatives that aim to address nutritional inequities facing racially-marginalized populations in the U.S [3, 47].

### Strengths & Limitations

Our study has strengths and limitations. The study population was a strength because it comprised a diverse sample of Black-identifying adults according to their age, sex, education level, socioeconomic status and geographic location. In addition, the online survey featured several valid and reliable screeners including the U.S. Food Security Module and the DSQ. The cross-sectional study design was a limitation because it did not permit the evaluation of causal or temporal associations. Longitudinal studies are needed to assess causal relationships between exposure to crime at food stores, food security, obesity, and FV consumption. All data were self-reported by survey respondents, which may have introduced reporting error or biases. Objective measures of body composition and more detailed measures of dietary intake are needed to improve upon this work. Finally, our study population comprised a volunteer sample of Black adults who are Cloud Research users. Our findings may not be applicable to all Black adults in the U.S. or other racial groups.

In summary, we documented associations between exposure to crime at local food stores, food insecurity, and obesity. These findings contribute novel information to the scientific literature on *(1)* the nutritional and health consequences of crime exposure at food stores in the U.S. and *(2)* the public health importance of community safety. Currently, there is a dearth of research that describes the mechanisms by which crime and violence, in any form (e.g., interpersonal, community, structural), negatively affect nutritional well-being [3]. To improve upon current understanding of nutritional inequities, especially those facing racially-marginalized populations, research is needed to further explore the interplay between important social determinants of health such as food access and community safety [3].
